# Can Spectral Power Be Used as a Candidate Seizure Marker of the Periodic Discharges Pattern?

**DOI:** 10.3389/fneur.2021.642669

**Published:** 2021-06-14

**Authors:** Jianhua Chen, Xiangqin Zhou, Liri Jin, Qiang Lu, Heyang Sun, Qing Liu, Yan Huang

**Affiliations:** Department of Neurology, Peking Union Medical College Hospital, Chinese Academy of Medical Sciences and Peking Union Medical College (CAMS & PUMC), Beijing, China

**Keywords:** spectrum analysis, EEG, seizure, frequency, marker

## Abstract

**Introduction:** It remains controversial whether the periodic discharges (PDs) pattern is an ictal or interictal phenomenon. The aims of the study are to apply time-frequency and power spectrum analysis to study the PDs pattern and prediction of seizures.

**Methods:** We retrospectively searched continuous electroencephalography (cEEG) recordings to identify patients exhibiting the PDs pattern. Artifact-free cEEG segments demonstrating the PDs pattern with stable baselines were chosen for time-frequency and power spectrum analysis.

**Results:** In total, 72 patients (1.3%) exhibited the PDs pattern, with a mean age 36.0 ± 20.7 years (range, 8–76 years). The median spectral power of PDs with a length of 60 s was 70.94 μV^2^ and that of PDs with a length of 20 s was 195.80 μV^2^. During follow-up, patients with spectral power of PDs of length 60 and 20 s lower than 28.65 and 36.09 μV^2^, respectively, exhibited no seizure. For predicting seizures, when the spectral power for PDs of 60 and 20 s equaled to 17.26 and 21.40 μV^2^, respectively, the diagnostic sensitivity was 100% and specificity was 86%. The locations of maximal spectral power of PDs, crude seizure onset zone (SOZ) judged from scalp EEG, and the most prominent regions of hyper- or hypo-metabolism on FDG-PET were congruent.

**Conclusions:** Spectral power might be a candidate seizure marker of the PDs pattern. High spectral power predicted a high risk of seizures, and low spectral power was associated with a low risk of seizures.

## Introduction

The use of continuous electroencephalography (cEEG) monitoring for the diagnosis of electrographic seizures or status epilepticus, and in patients with altered mental status with or without acute/chronic structural lesions, has grown tremendously in the last decade (1, 2). The periodic discharges (PDs) pattern is an abnormal finding on cEEG, observed in 8.6–14% of critically ill patients (1–4). Lateralized PDs reflected severe focal dysfunction and acute destructive processes, and generalized PDs could be related to toxic-metabolic encephalopathies and cerebral hypoxia. Up to now, it has remained controversial whether the PDs pattern is an ictal or an interictal phenomenon (5). Currently, there is no widely agreed-on standard of care for managing patients with PDs pattern epilepsy, and the use of aggressive therapy is still widely debated (5).

Time-frequency and power spectrum analysis is widely used in EEG signal analysis. To our knowledge, very few studies have applied signal analysis in studying the PDs pattern (6, 7). The current study sought to identify the occurrence of the PDs pattern and apply time-frequency and power spectrum analysis in studying the PDs pattern and prediction of seizures.

## Materials and Methods

### Patients

The study was conducted in the Department of Neurology in Peking Union Medical College Hospital, a tertiary hospital in Beijing, China, from October 2008 to October 2020. We retrospectively searched cEEG recordings to identify patients exhibiting the PDs pattern. The composition of the patients includes the ones admitted into general neurology ward, neurointensive care unit and epilepsy monitoring unit, who received cEEG monitoring. The original cEEG of the PDs pattern were visually identified by two of the authors (JHC and XQZ). In addition, we reviewed electronic medical records of all the patients for extraction of clinical information including demographic data, etiology for PDs, past and present medical history, level of consciousness (LOC), final diagnosis, anti-epileptic drugs (AEDs), prescriptions, follow-up clinical data, and neuroimaging.

### Definitions

We classified the PDs pattern according to the American Clinical Neurophysiology Society's standardized critical care EEG terminology (2013 version) (8, 9). Periodic was defined as repetition of a waveform with relatively uniform morphology and duration with a quantifiable inter-discharge interval between consecutive waveforms and recurrence of the waveform at nearly regular intervals. Discharges were defined as waveforms with no more than three phases (i.e., crossing the baseline no more than twice) or any waveform lasting 0.5 s or less, regardless of the number of phases (8, 9). A pattern was classified as rhythmic or periodic as long as it continued for at least six cycles (9). Duration of PDs was specified as the typical as well as the longest duration of a single occurrence of the pattern (8, 9). “Very long” meant equal to or over 1 h; “Long” 5–59 min; “Intermediate” 1–4.9 min; “Brief” 10–59 s; “Very brief” <10 s (8, 9). Prevalence of PDs was specified as percent of record occupied by the pattern (8, 9). “Continuous” meant ≥90% of record; “Abundant” 50–89% of record; “Frequent” 10–49% of record; “Occasional” 1–9% of record; “Rare” <1% of record (8, 9).

### Data Acquisition

cEEG data (Nihon Kohden Corporation), with an analog bandwidth of 0.5–70 Hz, at a sampling rate of 200 Hz (until September 2013) or 500 Hz (September 2013 and later), were acquired using 21 electrodes placed according to the International 10-20 System. Channel impedances were kept below 5 kΩ. Surface electromyography channels were added when necessary.

### EEG Data Analysis

#### EEG Data Preprocessing

Digital cEEG data were first converted to European Data Format (EDF) and imported into EEGLAB (10), an open source toolbox running in the MATLAB environment. All analyses were based on a referential montage of AV electrodes. An artifact-free cEEG epoch demonstrating the largest and clearest PDs pattern was segmented into epochs of 20 and 60 s, respectively, with zero time at the midpoint (6). The chosen epoch was at least 10 min before or after any clinical or electrographic seizures. The epochs presenting as (poly)spike-and-wave or sharp-and-wave (SW) complexes without interdischarge interval, or evolving discharges that reached more than 4 Hz, were not included in the study. EEG data was not band-pass filtered. This enabled low frequency periodic waves and PDs superimposed fast activity with each discharge can be seen. To eliminate line noise, 50-Hz notch filters were used. Independent Component Analysis algorithm (runica) was used for artifacts rejection (10).

#### Time-Frequency and Power Spectral Analysis

Time-frequency analysis was conducted using the short-term Fourier transform with a fixed 200 ms Hanning window (11). The data were low-pass filtered below 30 Hz. Power spectral analysis was conducted by the Fast Fourier Transform. The channel with the maximal magnitudes of power spectra was selected and the most prominent peak that possessed the frequency, which concluded from the time-frequency analysis, was identified to calculate the spectral power. Associated scalp topographies were then plotted.

### Statistical Analysis

Patients who exhibited the PDs patterns were divided into no seizure (*N* = 8) and seizure groups (*N* = 64), with the latter subdivided into status epilepticus (SE) (*N* = 13) and non-SE groups (*N* = 51). Descriptive statistics included age, frequencies and percentages for categorical variables, and means, medians and standard deviations for continuous variables. ANOVA was used for comparisons between groups for categorical variables with normal distributions. The Wilcoxon rank sum test and the Mann-Whitney *U*-test were used for comparison of continuous variables. Logistic multivariable regression was used to identify the relationship between waveforms of PDs and seizures. Statistical analyses were performed with SPSS Statistics 20.0 software (IBM, New York, USA). A *P*-value <0.05 was considered statistically significant.

## Results

### Demographic and Clinical Characteristics of Patients

In total, 5,740 consecutive patients underwent cEEG monitoring over the period, with most patients recorded for at least 24 h. Among the patients, 72 (1.3%) exhibited spontaneous PDs pattern, with a mean age of 36.0 ± 20.7 years (range, 8–76 years), including 40 males (55.6%) and 32 females (44.4%). LOC of patients included 68 (94.4%) cases of patients who were in an awake or lethargic state, and four (5.6%) cases of stupor or coma. There was no significant difference among the groups of no seizure, SE and non-SE groups in terms of age (*F* = 0.759, *P* = 0.472).

The etiology of the PDs pattern included three cases (4.2%) with acute ischemic cerebral infarction, 26 cases (36.1%) with central nervous system infection, 26 cases (36.1%) with epilepsy, seven cases (9.7%) with metabolic encephalopathy, three cases (4.2%) with autoimmune encephalitis, one case (1.4%) with brain tumor, one case (1.4%) with hypoxic-ischemic encephalopathy, one case (1.4%) with multiple sclerosis, and four cases (5.6%) with dementia. All the patients underwent head magnetic resonance imaging (MRI) examination, and among them, 41 patients (56.9%) exhibited structural lesions.

Fifty-one patients (70.8%) had clinical seizures during cEEG monitoring, including 27 patients (37.5%) suffering from focal onset seizures. Thirty-three patients (45.8%) received AEDs before cEEG, with two (2.8%) experiencing AEDs reduction, and 24 patients (33.3%) after cEEG. None of the patients were under sedation. Most patients were followed up for more than 6 months, with a mean follow-up period of 12.5 ± 20.5 months, except for one patient who died during hospitalization.

### Features of PDs Pattern

The mean frequency of PDs was 1.15 ± 0.62 Hz (range, 0.18–2.75 Hz). The typical duration of the PDs pattern was “Very long” in 16 patients (22.5%), “Long” in eight (11.3%), “Intermediate” in 17 (23.9%), “Brief” and “Very brief” in 32 (43.1%). The absolute amplitude of PDs was low in six patients (8.5%), medium in 56 patients (78.9%), and high in nine patients (12.7%). The PDs pattern in 36 patients (50.7%) had quasi-periodic characteristics. A plus modifier was found in 22 patients (30.6%). The prevalence of PDs was “Continuous” in 18 patients (25.0%), “Abundant” in 14 (19.4%), “Frequent” in 7 (9.7%), “Occasional” in 27 (37.5%), and “Rare” in 6 (8.3%).

### Relationships of Frequency, Amplitude, Duration, and Waveforms of PDs With Seizures

Binary logistic regression was used to identify factors related to seizures. The results showed that there were no associations between frequency, amplitude, duration, prevalence, polarity, number of phases, sharpness, triphasic morphology, plus or quasi- of PDs, and seizures (*P* > 0.1).

### Time-Frequency and Power Spectrum Analysis of PDs Pattern

Among the 72 patients exhibiting the PDs pattern, 71 patients' original cEEG recordings were included in the time-frequency and power spectrum analysis of the PDs pattern. In one patient the power spectrum was not computed, for the original cEEG recordings was missing.

**Figure 1:**
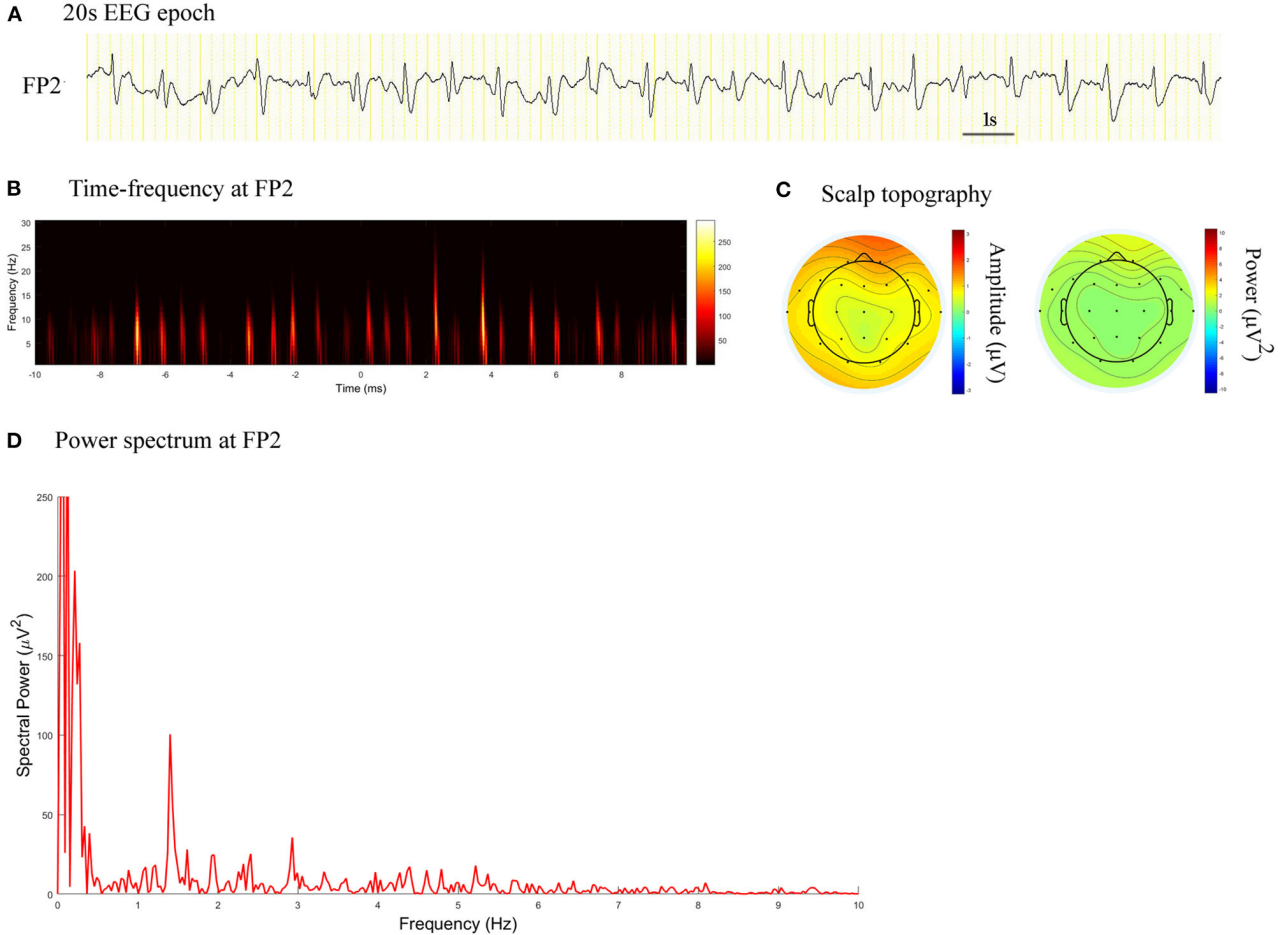
EEG epoch, time-frequency and power spectrum analysis, and scalp topography in a patient with CJD. **(A)** A 20 s EEG epoch at FP2 electrode. **(B)** Time-frequency analysis showed the PDs pattern had a frequency of 1.4 Hz. **(C)** Scalp topography showed the PDs pattern located in the right frontal pole. **(D)** Spectral power value for PDs of 20 s at 1.4 Hz was 100.5 μV^2^.

**Figure 2:**
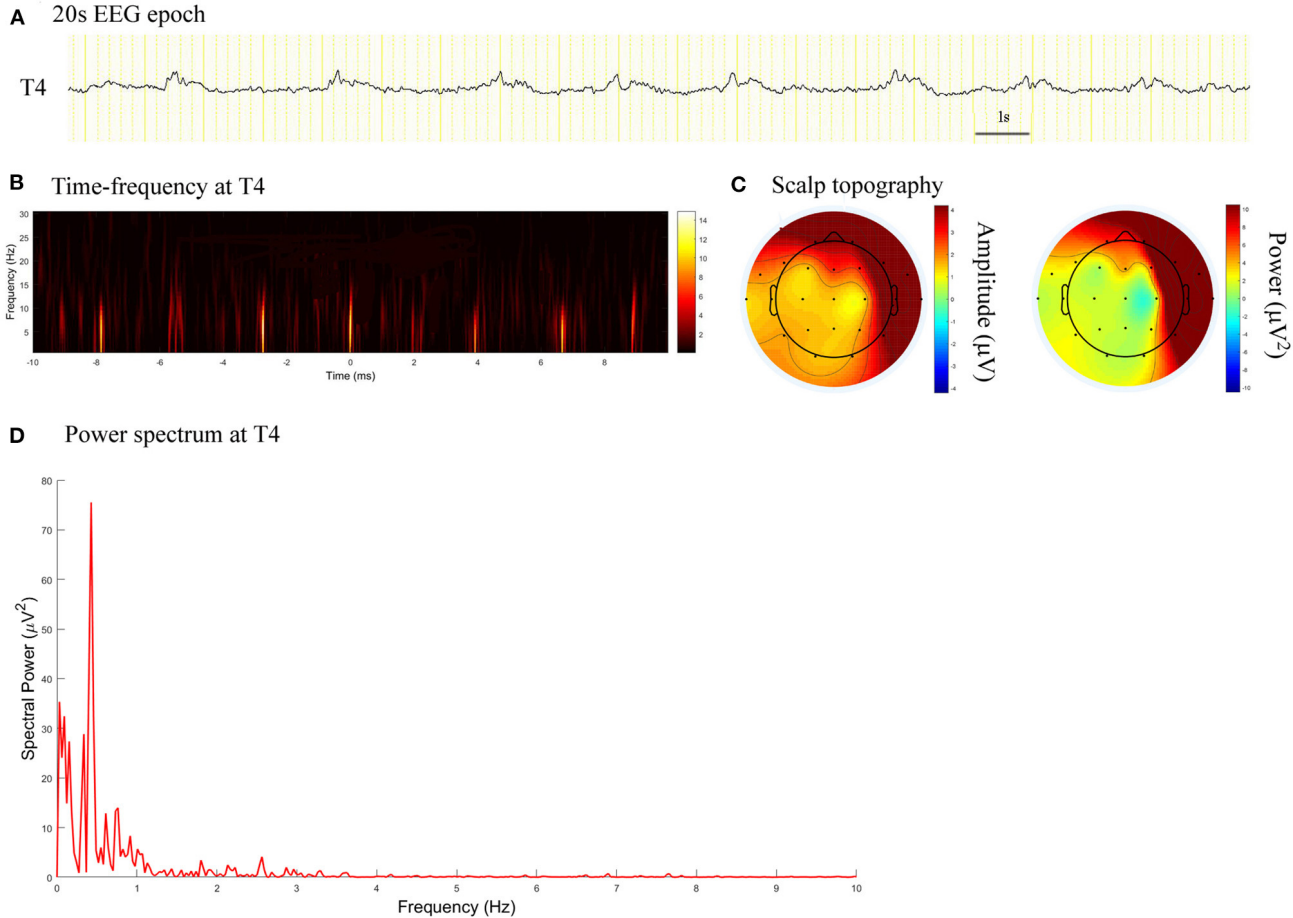
EEG epoch, time-frequency and power spectrum analysis, and scalp topography in a patient with acute viral encephalitis. **(A)** A 20 s EEG epoch at T4 electrode. **(B)** Time-frequency analysis showed the PDs pattern had a frequency of 0.4 Hz. **(C)** Scalp topography showed the PDs pattern located in the right temporal regions. **(D)** Spectral power value for PDs of 20 s at 0.4 Hz was 75.52 μV^2^.

The power spectrum of PDs demonstrated condensation and low-pass filtering characteristics (Figures 1, 2). The median spectral power of PDs with a length of 60 s was 70.94 μV^2^ (range, 3.95–60180.00 μV^2^), and that of PDs with a length of 20 s was 195.80 μV^2^ (range, 5.64–111400.00 μV^2^). During follow-up, patients with spectral power of PDs of length 60 and 20 s lower than 28.65 and 36.09 μV^2^, respectively, exhibited no seizure.

There were significant difference in spectral power between the no seizure and seizure groups, non-SE and SE groups, for PDs pattern activity of both 60 s and 20 s in duration (Z = −4.320, *P* = 0.000 and Z = −4.320, *P* = 0.000; Z = −4.030, *P* = 0.000 and Z = −4.297, *P* = 0.000).

### Comparison of Spectral Power of Length 60 and 20 s

**Figure 3:**
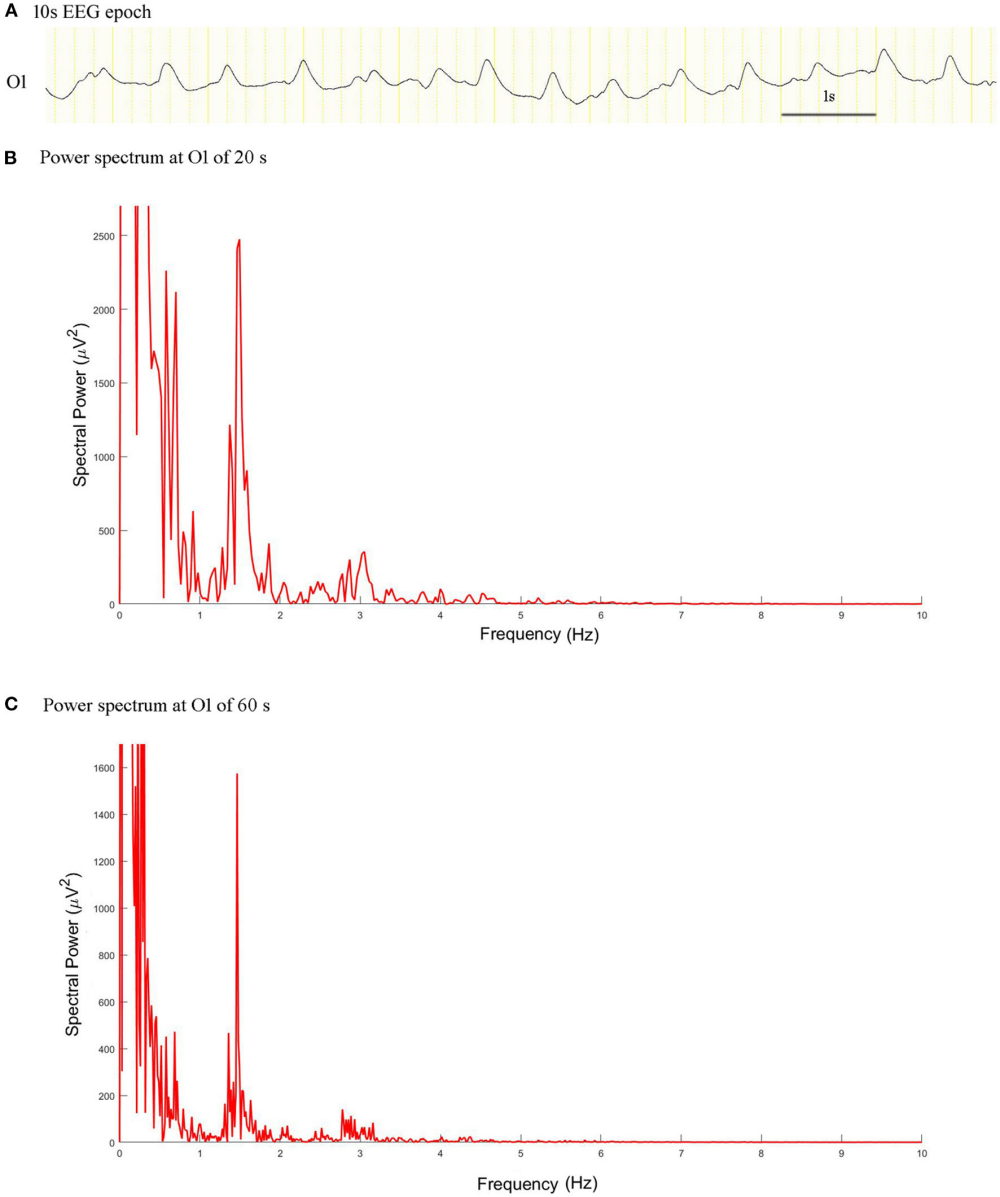
EEG epoch and power spectrum with a window of 20 and 60 s in a patient with intermediate duration PDs. **(A)** A 10 s EEG epoch at O1 electrode. **(B)** The spectral peaks of PDs of 20 s stood out of the background. **(C)** Power spectrum for PDs of 60 s exhibited more prominent condensation.

**Figure 4:**
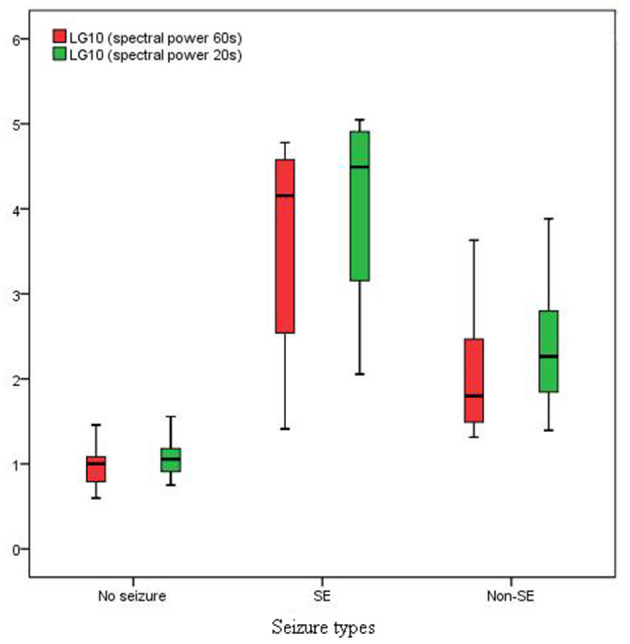
Spectral power in SE group was much higher than that in non-SE and no seizure groups (*P* < 0.01).

The spectral peak of PDs of 60 s in length was more clearly identified than that of 20 s in length, which stood out of the background with a very sharp shape (Figure 3). In the no seizure group, median spectral power was 10.05 μV^2^ of length 60 s and 11.38 μV^2^ of length 20 s. In the non-SE group, median spectral power was 62.97 μV^2^ of length 60 s and 183.60 μV^2^ of length 20 s. Median spectral power in the SE group was the highest, which was 14260 μV^2^ of length 60 s and 30950 μV^2^ of length 20 s (Figure 4).

There was no significant differences in spectral power between PDs of length 60 and 20 s in the no seizure group (Z = −1.859; *P* = 0.063). However, there were significant differences in spectral power between PDs of length 60 and 20 s in the non-SE group (Z = −4.930, *P* = 0.000) and SE group (Z = −2.271, *P* = 0.023).

### Threshold of Spectral Power for Predicting Seizures

The areas under the curve values of the receiver operating characteristic curve were 0.9710 (95% CI: 0.9149, 1.027; *P* < 0.0001) and 0.9866 (95% CI: 0.9586, 1.015; *P* < 0.0001) for spectral power of PDs of 60 and 20 s in length, respectively. When the spectral power for PDs of 60 s equaled to 17.26 μV^2^, (sensitivity, specificity) = (1, 0.86). When the spectral power for PDs of 20 s equaled to 21.40 μV^2^, (sensitivity, specificity) = (1, 0.86).

### Association of Location of PDs With Glucose Metabolism Measured by ^18^F-Fluorodeoxyglucose Positron Emission Tomography (FDG-PET)

**Figure 5:**
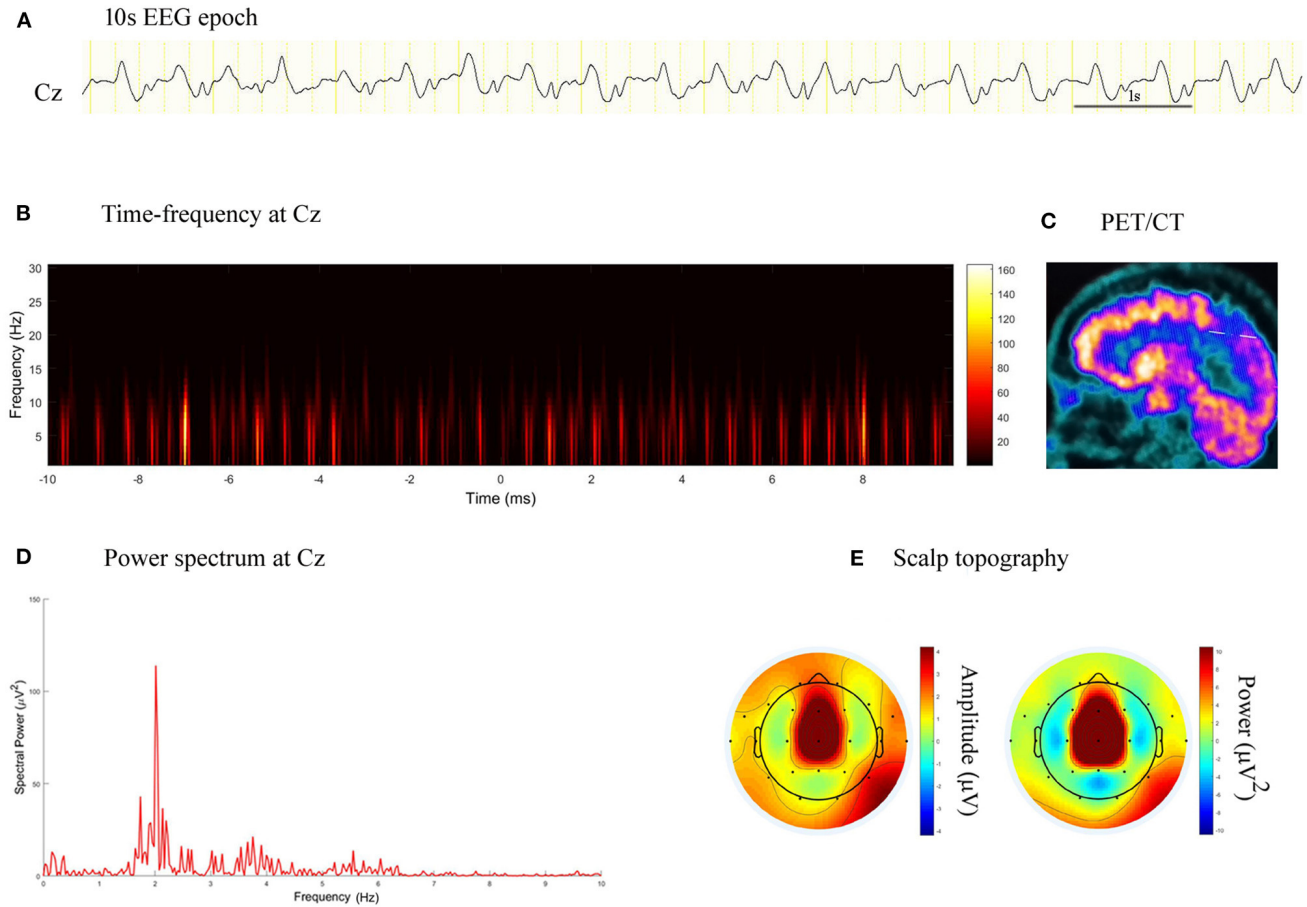
EEG epoch, time-frequency and power spectrum analysis, and PET/CT scan in a patient with stroke. **(A)** A 10 s EEG epoch at Cz electrode. **(B)** Time-frequency analysis showed PDs had a frequency of 2.0 Hz. **(C)** PET/CT scan demonstrated decreased FDG uptake in the right paracentral lobule. **(D)** Spectral power value for PDs of 20 s at 2.0 Hz was 113.90 μV^2^. **(E)** Scalp topography showed the PDs pattern located in the midline central.

Fourteen patients (19.4%) had FDG-PET scan. Among them, 12 patients underwent FDG-PET scan during contemporaneous PDs, and one underwent a scan before the PDs period and one after the PDs period. Three patients exhibited markedly increased focal metabolism, and 11 exhibited hypometabolism (Table 1). The location of maximal spectral power of PDs, crude seizure onset zone (SOZ) judged from scalp EEG, and the most prominent region of hyper- or hypo-metabolism on FDG-PET were congruent (Figure 5).

**Table 1:** Manifestation of FDG-PET in patients with the PDs pattern.

**Case**	**Type of PDs**	**Frequency of PDs**	**Period of PET and PDs**	**Manifestation of PET**	**Seizure**	**Etiology**	**Locations of PDs and PET are congruent**
1	GPDs	1.71 Hz	Within	Multiregional hypermetabolism	SE	CJD	Yes
2	BIPDs	0.85 Hz	Within	Multiregional with regional intense hypometabolism	Focal seizure	Epilepsy	Yes
3	LPDs	0.40 Hz	Within	Regional hypometabolism	Focal seizure	Epilepsy	Yes
4	GPDs	2.44 Hz	Within	Multiregional hypometabolism	Focal seizure	Epilepsy	Yes
5	LPDs	0.21 Hz	Within	Regional hypometabolism	Focal seizure	Epilepsy	Yes
6	LPDs	0.44 Hz	Within	Regional hypermetabolism	Focal seizure	Acute viral encephalitis	Yes
7	GPDs	1.47 Hz	Within	Diffused with regional intense hypometabolism	Myoclonus	CJD	Yes
8	LPDs	1.56 Hz	Within	Regional hypermetabolism	Myoclonus	Tumor	Yes
9	LPDs	0.43 Hz	Within	Diffused hypometabolism	None	Dementia	Yes
10	GPDs	2.01 Hz	Within	Regional hypometabolism	SE	Stroke	Yes
11	LPDs	0.99 Hz	Within	Regional hypometabolism	Focal seizure	Multiple sclerosis	Yes
12	LPDs	0.67 Hz	Within	Regional hypometabolism	Focal seizure	Stroke	Yes
13	GPDs	1.53 Hz	Before	Multiregional with regional intense hypometabolism	None	Dementia	Yes
14	LPDs	1.77 Hz	After	Diffused with regional intense hypometabolism	Focal seizure	Autoimmune encephalitis	Yes

*FDG-PET, ^18^F-fluorodeoxyglucose positron emission tomography; PDs, periodic discharges; GPDs, generalized periodic discharges; BIPDs, bilateral independent periodic discharges; LPDs, lateralized periodic discharges; SE, status epilepticus; CJD, Creutzfeldt-Jakob disease*.

## Discussion

To date, there is no definitive consensus regarding whether PDs (generalized, lateralized, or bilaterally independent) represent an epiphenomenon of acute neuronal injury (essentially harmless) or an ictal phenomenon (causing harm and potentially warranting intervention), or both (12). The current results revealed that spectral power of PDs was helpful for predicting the risk of seizures. For PDs of 60 or 20 s in length, spectral power values over 17 or 21 μV^2^ indicated a high risk of seizures and might be taken as a sensitive marker for near-term seizures if no intervention was carried out.

### Occurrence of PDs

The results revealed that the occurrence of the PDs pattern in the patients who received cEEG monitoring was 1.3%, which was less than the prevalence of 8.6–13.9% among critically ill patients in previous literature (3, 4). The difference between the current study and previous reports indicated that, although, PDs were usually seen in patients with serial seizures and acute structural brain lesions, they were more common in patients with altered mental status. In the current study, the participants were outside intensive care unit (ICU), and only 5.6% of them were in a mental state of stupor or coma, whereas, the patients in previous studies were from ICU, and stupor/coma ones accounted for 31–51% of the sample (2, 4).

### Preponderance of Time-Frequency and Power Spectrum Analysis in PDs

PDs do not constitute a specific pattern of neurological disease (13). Associations between PDs and seizure have been studied in critically ill adult patients (1, 4). Newey et al. (1) found that sharply contoured morphology, overlying fast frequencies, or rhythmicity, exhibited a progressively higher risk of seizures on cEEG, whereas, blunt delta morphology had the lowest risk of seizures. Rodriguez Ruiz et al. (4) reported that lateralized PDs were associated with seizures when the frequency was 1.5 Hz or faster, or when they were associated with a plus modifier. However, previous studies usually combined PDs, rhythmic delta activity (RDA), and evolution discharges to analyze the associations among them (1, 2, 7). These studies were all based on observation of EEG data using the naked eye, potentially causing identification errors and lower inter-rater reliability (1). In the current study, (poly)spike-and-wave or sharp-and-wave (SW) complexes without interdischarge interval, and evolving discharges that reached more than 4 Hz were excluded because these types of patterns are more likely be related to a risk of seizures. RDA was also not included, providing a more specific examination of the PDs pattern. In our study, two patients exhibited generalized or lateralized PDs with a frequency over 1.5 Hz, both of whom had never suffered from seizures during long-term follow-up. Overall, signal analysis of EEG data was more objective and quantitative than observation with the naked eye. By means of time-frequency and power spectrum analysis, the spectral peak of PDs could be clearly identified and precisely located, which might help to predict the risk of seizures more accurately than by observing cEEG with the naked eye.

### Value of Spectral Power of PDs in Prediction of Seizures

The pathophysiological and clinical significance of PDs has traditionally been unclear (14). The current results suggested that spectral power might be used as a candidate seizure marker of the PDs pattern, to identify whether it had enough strength to produce clinical symptoms or not. Patients with high spectral power of PDs exhibited an ictal condition, whereas, a very high value predicted SE, indicating that vigorous therapy should be considered. Patients with a low spectral power of PDs exhibited an interictal state or had no relationship with seizures, suggesting that AEDs might not be required.

### Priority of Power Spectrum of 60 and 20 s PDs

Because the typical durations of PDs pattern were over 60 s in 57% patients, and between 20 and 60 s in 38%, we chose 20 and 60 s as the window lengths in spectrum computing. The current results revealed that the frequency of PDs was more accurately quantified using time-frequency and power spectrum analysis. A power spectrum displayed the coefficients for each frequency measured by a fast Fourier transform as a graph of power values (15). In general, due to the relationship between fast Fourier transform spectral resolution and window length, high spectral resolution can only be achieved with relatively long windows, but this inevitably results in a loss of temporal resolution (16). This result explained why the power spectrum of 60 s PDs exhibited more prominent condensation and low-pass filtering characteristics than 20 s PDs. In the non-SE and SE groups, there was a difference in spectral power between PDs of 60 and 20 s, because in these groups, the duration of the PDs pattern varied from brief to very long, and the frequency changed between a static and a fluctuating state. We thought the spectral power of PDs with an epoch of 20 s was better in predicting the risk of seizures than that with an epoch of 60 s, when duration of PDs pattern was brief. Spectral power of 60 s might be preferred in patients with an intermediate, long, or very long duration, considering the more prominent condensation and higher spectral resolution. Kalamangalam and Slater (6) also found condensed and low-pass filtering dynamics characteristics of the PDs pattern, reporting that higher harmonics in the PDs spectrum can arise *via* recurrent feedback, possibly from entrained single units.

### Value of Combination of the Location of PDs and Metabolic Activity on FDG-PET

The location of the maximal power spectrum of PDs might potentially help to estimate crude SOZ, particularly when combined with ictal EEG and FDG-PET scan. The PDs pattern could demonstrate hyper- or hypo-metabolism in concurrent FDG-PET scans. Patients with Creutzfeldt-Jakob disease (CJD) typically exhibited diffuse hypometabolism on FDG-PET, whereas, intense hypometabolism might be observed in focal areas, as in one patient in our study who exhibited regional occipital cortex hypometabolism highlighted within diffuse cerebral cortex hypometabolism. In addition, we found that CJD patients displayed hypermetabolism on FDG-PET when experiencing SE. Focal hyper- or hypo-metabolism on FDG-PET associated with PDs suggested that PDs might be helpful for identifying crude SOZ. It has been reported that metabolic activity, as defined by the uptake of FDG on PET, took advantage of the physiological preferential use of anaerobic glycolysis over oxidative phosphorylation (17). Several case reports and studies about metabolic characteristics of PDs have suggested that FDG-PET could be a candidate metabolic marker of electrographic SE or electroclinical SE (16–20). Newey et al. (18) reported one case presenting with hypermetabolism on FDG-PET and PDs in the same hemisphere. Handforth et al. (14) reported that intense focal hypermetabolism was associated with PDs and dissipated when PDs decreased. Ergün et al. (19) reported a case of PDs by single photon emission computed tomography (SPECT) study. They found increased cerebral blood flow during PDs on EEG and decreased perfusion in the corresponding region after the PDs disappeared. Lee and Schauwecker (20) also reported that SPECT results in one patient with PDs revealed increased regional cerebral perfusion in the PDs focal region. Struck et al. (21) studied 18 subjects with FDG-PET during PDs and RDA patterns. FDG-PET revealed hypermetabolic findings in 61% of patients, hypometabolic findings in 28% of patients, and normal findings in 11% of patients. Subramaniam et al. (22) examined nine patients, reporting that metabolic activity increased monotonically with lateralized PDs frequency. The researchers proposed that lateralized PDs frequency should be a measure of interest when developing neuroprotection strategies. In this study, we found no correlation between metabolic activity of PDs focus and PDs frequency. One patient, whose PDs frequency was 0.44 Hz, had regional hypermetbolism on FDG-PET scan. Two patients, whose PDs frequencies were 2.44 and 2.01 Hz, respectively, showed multiregional hypometabolism on FDG-PET. The current results implied feasibility of applying spectrum power of PDs in predicting seizures and forecasting crude SOZ from scalp EEG. In addition, our findings suggested that metabolic manifestations in a PDs focus was not always monotonically associated with PDs frequency, but etiology and concomitant SE also having effects and spectral power of PDs playing an important role.

### Limitations and Outlook

We made a hypothesis that the more spectral power the PDs pattern had, the more likely it could produce epileptic seizures. However, the current study involved several limitations, including the retrospective study design and small number of participants. A prospective study with more patients would be helpful for confirming the hypothesis. Furthermore, the interpretation was posteriori which was difficult to be done in real time. In the future, the development of algorithms that could detect the pattern in a specific power could help to increase detection of seizures. Also, the use of the spectral analysis at bedside, could lead to recognition of the pattern tending to induce seizures and prompt treatment. As an additional limitation, all of the data analyzed in the current study were from scalp EEG. We tried to make an assumption that the location of the maximal power spectrum of PDs might potentially help to estimate crude SOZ, particularly when combined with ictal EEG and FDG-PET scan, while EEG data from intracranial electrodes would be better for identifying SOZ and its relationship with the location of maximal power spectrum of PDs. Considering the small sample size, the threshold of spectral power for predicting seizures was only a rough estimate. Larger datasets from more centers will be needed to study the association between PDs spectral power and risk of seizures.

## Conclusions

The current results suggested that spectral power might be used as a candidate seizure marker of the PDs pattern. High spectral power indicated a high risk of seizures, and very high values were common in patients with SE. Low spectral power was associated with a low risk of seizures.

## Data Availability Statement

The data analyzed in the study is available on reasonable request. Requests to access the datasets should be directed to deweiyy@163.com.

## Ethics Statement

The studies involving human participants were reviewed and approved by Human Research Ethics Committee of Peking Union Medical College Hospital. Written informed consent to participate in this study was provided by the participants' legal guardian/next of kin.

## Author Contributions

JC designed the study. JC, XZ, LJ, QLu, HS, QLi, and YH acquired the data. JC and XZ preprocessed the data, performed the data analysis, and drafted the manuscript. All the authors contributed to the article and approved the submitted version.

## Conflict of Interest

The authors declare that the research was conducted in the absence of any commercial or financial relationships that could be construed as a potential conflict of interest.
